# Toll-Like Receptor 1/2 and 5 Ligands Enhance the Expression of Cyclin D1 and D3 and Induce Proliferation in Mantle Cell Lymphoma

**DOI:** 10.1371/journal.pone.0153823

**Published:** 2016-04-28

**Authors:** Katy Mastorci, Elena Muraro, Elisa Pasini, Chiara Furlan, Luca Sigalotti, Marina Cinco, Riccardo Dolcetti, Elisabetta Fratta

**Affiliations:** 1 Cancer Bio-Immunotherapy Unit, Department of Translational Research, Centro di Riferimento Oncologico, IRCCS—National Cancer Institute, Aviano (PN), Italy; 2 Princess Margaret Cancer Centre, University Health Network and TECHNA Institute for the Advancement of Technology for Health, TMDT, Room 11–314, 101 College Street, Toronto, ON M5G 1L7, Canada; 3 Spirochete Laboratory, Department of Life Sciences, University of Trieste, Trieste, Italy; 4 University of Queensland Diamantina Institute, Translational Research Institute, Brisbane, Australia; University of Salerno, Faculty of Medicine and Surgery, ITALY

## Abstract

Mantle cell lymphoma (MCL) is an aggressive B-cell non-Hodgkin’s lymphoma with a still undefined etiology. Several lines of evidence are consistent with the possible involvement of peculiar microenvironmental stimuli sustaining tumor cell growth and survival, as the activation of Toll-like receptors (TLR) 4 and 9. However, little is known about the contribution of other TLRs of pathogenic relevance in the development of MCL. This study reports evidence that MCL cell lines and primary MCL cells express different levels of TLR2 and TLR5, and that their triggering is able to further activate the Akt, MAPK, and NF-κB signaling cascades, known to be altered in MCL cells. This leads to the enhancement of cyclin D1 and D3 over-expression, occurring at post-translational level through a mechanism that likely involves the Akt/GSK-3α/β pathway. Interestingly, in primary B cells, TLR1/2 or TLR5 ligands increase protein level of cyclin D1, which is not usually expressed in normal B cells, and cyclin D3 when associated with CD40 ligand (CD40L), IL-4, and anti-human-IgM co-stimulus. Finally, the activation of TLR1/2 and TLR5 results in an increased proliferation of MCL cell lines and, in the presence of co-stimulation with CD40L, IL-4, and anti-human-IgM also of primary MCL cells and normal B lymphocytes. These effects befall together with an enhanced IL-6 production in primary cultures. Overall, our findings suggest that ligands for TLR1/2 or TLR5 may provide critical stimuli able to sustain the growth and the malignant phenotype of MCL cells. Further studies aimed at identifying the natural source of these TLR ligands and their possible pathogenic association with MCL are warranted in order to better understand MCL development, but also to define new therapeutic targets for counteracting the tumor promoting effects of lymphoma microenvironment.

## Introduction

Mantle cell lymphoma (MCL) is a distinct entity accounting for 3–10% of non-Hodgkin lymphomas characterized by advanced stage at presentation and aggressive clinical behaviour, with poor response to conventional therapeutic regimens and an often dismal prognosis.[1,2] A subset of MCL, however, shows an indolent clinical course and a long survival, often not even requiring chemotherapy for long periods.[3,4] More than 95% of MCLs carry the t(11;14)(q13;q32) translocation, which results in a juxtaposition of the *CCND1* gene locus to the immunoglobulin heavy chain promoter and the subsequent cyclin D1 over-expression,[1,5] leading to the deregulation of the cyclin D/Rb pathway. Cyclin D1 over-expression, however, is not sufficient for lymphomagenesis,[1,2] and cooperation with still poorly defined microenvironmental stimuli, as well as additional genetic changes are required to induce and sustain the transformed phenotype of MCL cells.[1,2]

Several lines of evidence support a pathogenic relevance of tumor microenvironment in MCL. It is noteworthy that MCL often involves (or even presents at) extra-nodal sites, mainly Waldayer’s ring and the gastrointestinal tract,[1,5] where factors present in these districts could promote lymphoma cell growth and survival. Moreover, CD40 activation was shown to promote primary MCL cell proliferation, which is further enhanced by IL-4 or IL-10 co-stimulation.[6–8] Recent findings also demonstrated that IL-6 plays a critical role in promoting MCL cell growth, survival and drug resistance.[9] Identification of microenvironmental factors critical for MCL may be relevant not only to improve our knowledge on MCL pathogenesis, but it may also favor the exploitation of new therapeutic targets.

Chronic inflammation is known to provide a favorable milieu for lymphomagenesis by promoting local production of a variety of factors able to stimulate the growth and survival of lymphoid cells while inhibiting antitumor immune responses.[10,11] A relevant role in this process is played by pathogen-associated molecular patterns (PAMPs), molecules recognized by Toll-like receptors (TLRs), transmembrane receptors expressed by immune cells behaving as key sensors of a variety of PAMPs from bacteria, virus and fungi, and representing crucial regulators of both innate and adaptive immune responses against pathogen infection. TLRs can also recognize and be activated by still poorly defined endogenous ligands.[10,12,13] Accumulating evidence however indicates that functional TLRs are also expressed by a wide variety of malignancies, including lymphomas, and activation of tumor TLRs was shown to promote neoplastic cell proliferation, resistance to apoptosis and production of immunosuppressive cytokines.[10,14]

B-cell malignancies show heterogeneous expression of TLRs and a variable pattern of response to TLR activation. In particular, MCL cells were shown to express TLR9, the receptor for CpG motifs within microbial DNA, and to respond with activation and enhanced proliferation when stimulated with CpG oligodeoxynucleotides.[15] Moreover, activation of TLR4 signaling by lipopolysaccharide was able to induce MCL cell growth and up-regulate production of IL-6, IL-10, and VEGF.[16] Nevertheless, MCL cells may variably express several other TLRs,[16] whose triggering by microenvironmental factors might contribute to the complex pathogenesis of this lymphoma. At present, little is known about the role of TLR2 and TLR5 in MCL, although their expression has been frequently reported to exhibit tumor-promoting signaling rather than antitumor responses. Indeed, TLR2 and TLR5 were shown to regulate tumor tolerance, cancer progression and metastasis, although with effects that may vary according to cancer cell type.[10,14,17] TLR2 can form heterodimers with TLR1 or TLR6 to recognize diacylated and triacylated bacterial lipoproteins,[18] whereas TLR5 is a receptor for flagellin, a component of bacterial flagella.[19] Therefore, local infections by microorganisms producing PAMPs activating these TLRs could influence the growth and survival of MCL cells, particularly at distinct anatomic sites. Interestingly, TLR2 and TLR5 variants have been identified with increased risk of several tumors, including MALT-lymphoma.[20] Furthermore, it has been recently demonstrated that TLR2 mutations may contribute to the pathogenesis of a subset of MCL by modulating tumor microenvironment responses.[21] Thus, aiming to identify new therapeutic targets for counteracting the tumor promoting effects of lymphoma microenvironment, we extensively investigated the role of TLR2 and TLR5 and their signaling in MCL cells.

In the present study, we provide novel data showing that MCL cell lines and primary MCL cells constitutively express different levels of TLR2 and TLR5. We also demonstrate that their triggering may enhance the inherent activation of Akt, MAPK, and NF-kB signaling pathways and up-regulate cyclin D1 and D3 expression at post-translational level. Moreover, we show that TLR1/2 or TLR5 recombinant ligands are able to increase the expression of cyclin D1 and cyclin D3 in normal B lymphocytes co-stimulated by CD40L, IL-4, and anti-human-IgM. Signaling by TLR1/2 and TLR5 results in increased proliferation of MCL cell lines and, in the presence of the co-stimuli, of primary MCL cells and normal B lymphocytes. These results may support the hypothesis of a contribution of TLRs endogenous or exogenous ligands in the acquisition of a B cell malignant phenotype during MCL development.

## Material and Methods

### Primary B cells

Primary B cells were negatively isolated from healthy donor PBMCs (n = 4) using the B-cell isolation kit (Miltenyi Biotech) and cultured in RPMI-1640 (Gibco), containing 2 mM L-glutamine, 20% fetal bovine serum (FBS; Gibco), 100 μg/ml streptomycin and 100 IU/ml penicillin (Sigma-Aldrich), at 37°C in 5% CO_2_. Lymphoma cells from six MCL patients (median age 58, range 50–76 years; all male) affected by MCL in leukemic phase (>90% CD19^+^ cells) were also used. Written informed consent was obtained from all patients and donors. The study was reviewed and approved by the Internal Review Board (IRB) of the Centro di Riferimento Oncologico CRO (IRCCS) of Aviano (IRB-03-2010). Cells were activated with soluble CD40L (Alexis, 1 μg/ml), 1 μg/ml of the corresponding enhancer (Enzo Life Sciences), 10 ng/ml of IL-4 (R&D Systems), and 2 μg/ml F(ab’)_2_ fragment Goat Anti-Human IgM (Jackson ImmunoResearch), and treated for 72 or 96 hours with 30 ng/ml Pam_3_CSK_4_ (TLR1/2 ligand; Imgenex), or 75 ng/ml purified flagellin (TLR5 ligand; Imgenex).

### Cell lines

Mino, SP53, and Jeko-1 cell lines were contributed by Dr. Raymond Lai. Cell lines were authenticated by fingerprinting (Power Plex 1.2, Promega) in January 2012. All cells were cultured in RPMI-1640 supplemented with 10% FBS heat-inactivated, 100 U/ml penicillin, 100 μg/ml streptomycin and 20 mM L-glutamine. Proliferation experiments were performed after an overnight starvation (2% FBS), with or without 30 ng/ml TLR1/2 ligand, or 150 ng/ml TLR5 ligand.

### Flow cytometry analysis

The following fluorescent-conjugated antibodies were used: α-TLR2/CD282 fluorescein isothiocyanate (FITC; mouse IgG2a; clone TL2.1), α-TLR5 FITC (mouse IgG2a; clone 85B152.5) from Imgenex; α-TLR2/CD282-FITC (mouse IgG2a; clone TL2.1), α-Ki-67-Phycoerythrin (PE; mouse IgG1; clone 20Raj1) from eBioscience; α-CD20 R Phycoerythrin-Cyanin 5.1 (PeCy5) from Beckman Coulter. A FITC-conjugated mouse IgG2a isotypic antibody (Becton Dickinson) was used as negative control. All antibodies were used in an appropriate volume of 10% rabbit serum (Dako) and PBS to reduce unspecific signal. To evaluate intracellular Ki-67 expression, cells were fixed with 2% paraformaldehyde in complete medium for 10 minutes at room temperature, washed in complete medium and permeabilized using 90% methanol diluted in H_2_O. After 10 minutes incubation in ice, cells were washed in permeabilization buffer (PBS with 0.5% BSA; Sigma-Aldrich) and stained with 0.06 μg of α-Ki67 antibody in 100 μl of 2% rabbit serum diluted in permeabilization buffer at 4°C for 30 minutes. Samples were washed twice and re-suspended in PBS with 1% paraformaldehyde for flow cytometry analysis. Proliferation of primary B lymphocytes was performed by carboxyfluorescein diacetate, succinimidyl ester (CFSE; Molecular Probes) dilution. Briefly, primary B cells (2x10^6^/ml) were stained in PBS with 0.5 μM CFSE for 15 minutes at 37°C, washed once and maintained in complete medium for further 30 minutes at 37°C. Cells were finally washed twice and cultured 1x10^6^/ml in complete medium (20% FBS) with TLR ligands. After 72 or 96 hours cells were collected, washed in PBS, and stained with 1μg/ml of Propidium Iodide in PBS, to exclude dead cells. Cytofluorimetric analysis was performed with a Cytomics FC500 (Beckman Coulter), acquiring at least 5x10^3^ cells for surface and Ki-67 staining, and 10x10^3^ Propidium Iodide-negative cells for CFSE labelling. Data were analyzed with CXP (Beckman Coulter) and FlowJo software (Tree Star, Inc., Ashland, OR, USA).

### IL-6 detection

IL-6 levels were assessed in triplicate samples using the Human IL-6 ELISA Kit (Thermo Scientific) according to manufacturer’s instructions. Absorbance was determined at 450±10 nm with a microtiter plate reader (Bio-Tek Instruments).

### Real-time quantitative RT-PCR analysis

Total RNA was prepared using TriZol reagent (Invitrogen) and digested with RNase-free DNase I (Roche) to remove contaminating genomic DNA. One μl of DNase I reaction buffer (200 mM Tris-HCl pH 8, 500 mM KCl, 20 mM MgCl_2_) and 10 units of DNase I (10U/μl) were added per μg of RNA sample in 10 μl reaction volume. After incubation at 37°C for 30 minutes, DNase I was inactivated by heating at 65°C for 10 minutes in presence of 25 mM EDTA (1 μl/μg of RNA). cDNA synthesis was performed on 1 μg total RNA using MMLV reverse transcriptase (Invitrogen) and random hexamer primers (Promega). Gene expression measurement was performed with the ABI prism 7700 Sequence Detection System (Applyed Biosystems). cDNA standards were obtained by RT-PCR amplification of the specific mRNAs and quantitated by NanoDrop® ND-1000 UV-Vis Spectrophotometer. SYBR-green quantitative RT-PCR reactions were performed on 20 ng of retrotranscribed total RNA in a final volume of 25 μl 1 X SYBR Green Master Mix (Applied Biosystems) at 95°C for 10 min, followed by 45 cycles at 95°C for 15 s and at 60°C for 1 min, followed by dissociation performed at 95°C for 15 s, 60°C for 20 s and 95°C for 15 s. SYBR-green primers sets were as follows: β-actin, forward CGA GCG CGG CTA CAG CTT and reverse CCT TAA TGT CAC GCA CGA TT; cyclin D1, forward GTGCTGCGAAGTGGAAACC and reverse ATCCAGGTGGCGACGATCT; cyclin D3, forward GACCATCGAAAAACTGTGCATCTA and reverse CCCACTTGAGCTTCCCTAGGA; IL-6, forward TACATCCTCGACGGCATCTC and reverse ACCAGGCAAGTCTCCTCATTG. The copy number of specific and β-actin genes was established in each sample by extrapolation of the standard curve. The number of selected gene cDNA molecules in each sample was then normalized to the number of cDNA molecules of β-actin.

### Extract preparation and Western Blot analysis

Whole-cell lysates were prepared in lysis buffer (50 mm Tris-HCl, pH 7.5; 150 mm NaCl; 2 mm EDTA; 2 mm EGTA; 2 mm sodium orthovanadate; 25 mm -glycerolphosphate; 25 mm NaF; 1 mm PMSF; 1 m okadaic acid; 5 g/ml leupeptin; 5 g/ml aprotinin; 0.2% Triton X-100; 0.3% NP40) and lysed for 30 min on ice. Total protein extracts were obtained by centrifugation at 16,000 g for 30 min and protein concentration was determined by Bio-Rad Bradford Protein Assay. For nuclear and cytoplasmic extract preparation, cells were incubated in ice-cold cell lysis buffer (10 mm HEPES pH 7.9, 10 mm KCl, 0.1 mm EDTA, 0.1 mm EGTA, 1 mmDTT, 0.5 mm PMSF, 5 g/ml leupeptin, 1 g/ml aprotinin, 1 m okadaic acid, 0.1% NP40). Incubation was continued for 15 min, checked microscopically for cell lysis and then centrifuged at 600 g for 5 min at 4°C. The supernatant (cytoplasmic fraction) was recovered and the pellet (nuclear fraction) was re-suspended in ice-cold extraction buffer (20 mm HEPES, 400 mm NaCl, 1 mm EDTA, 0.5 mm PMSF, 5 g/ml leupeptin, 1 g/ml aprotinin, 1 m okadaic acid, 1% NP40) and lysed for 2 h on ice. Nuclear extracts were clarified at 16,000 g for 15 min. Immunoblotting was performed using the enhanced chemiluminescence plus detection system (PerkinElmer) through Chemidoc XRS+ instrument (Biorad). Criterion™ and Mini-Protean® TGX Stain Free™ gels from Biorad were used to succeed in gel imaging for protein loading control. Phospho-Akt (Ser473), phospho-glycogen synthase kinase-3beta (GSK3-β) (Ser21/9), phospho-NF-κB p65 (Ser536), NF-κB p105/p50, NF-κB p100/p52, phospho-p38 (Thr180/Tyr 182), phospho-Erk1/2 (Thr202/Tyr204), and Cyclin D3 (DCS22) antibodies were from Cell Signaling Technology; β-tubulin (H-235), cyclin D1 (DCS-6), cyclin D3 (C-16), p65 (F6) were from Santa Cruz Biotechnology; topo II was from BD trasduction laboratories. TLRl/2 and TLR5 ligands were used at 50 ng/ml.

### Statistical evaluations

B-cell proliferation was converted through the “Proliferation tool” of the FlowJo software (Tree Star, Inc., Ashland, OR, USA) in % Divided, which represents how many cells divided at least once. The increase in Ki-67 expression was defined by a ratio between the Ki-67 Mean Fluorescence Intensity (MFI) of treated cells and the same parameter detected in untreated cells. The Student’s t test for two-tailed distributions was used for the analysis of triplicates in the IL-6 ELISA assay and for the comparison of 3 independent proliferation experiments. Data were considered statistically significant when p ≤ 0.05 (two-sided).

## Results

### TLR2 and TLR5 expression in normal B lymphocytes and MCL cells

**Fig 1:**
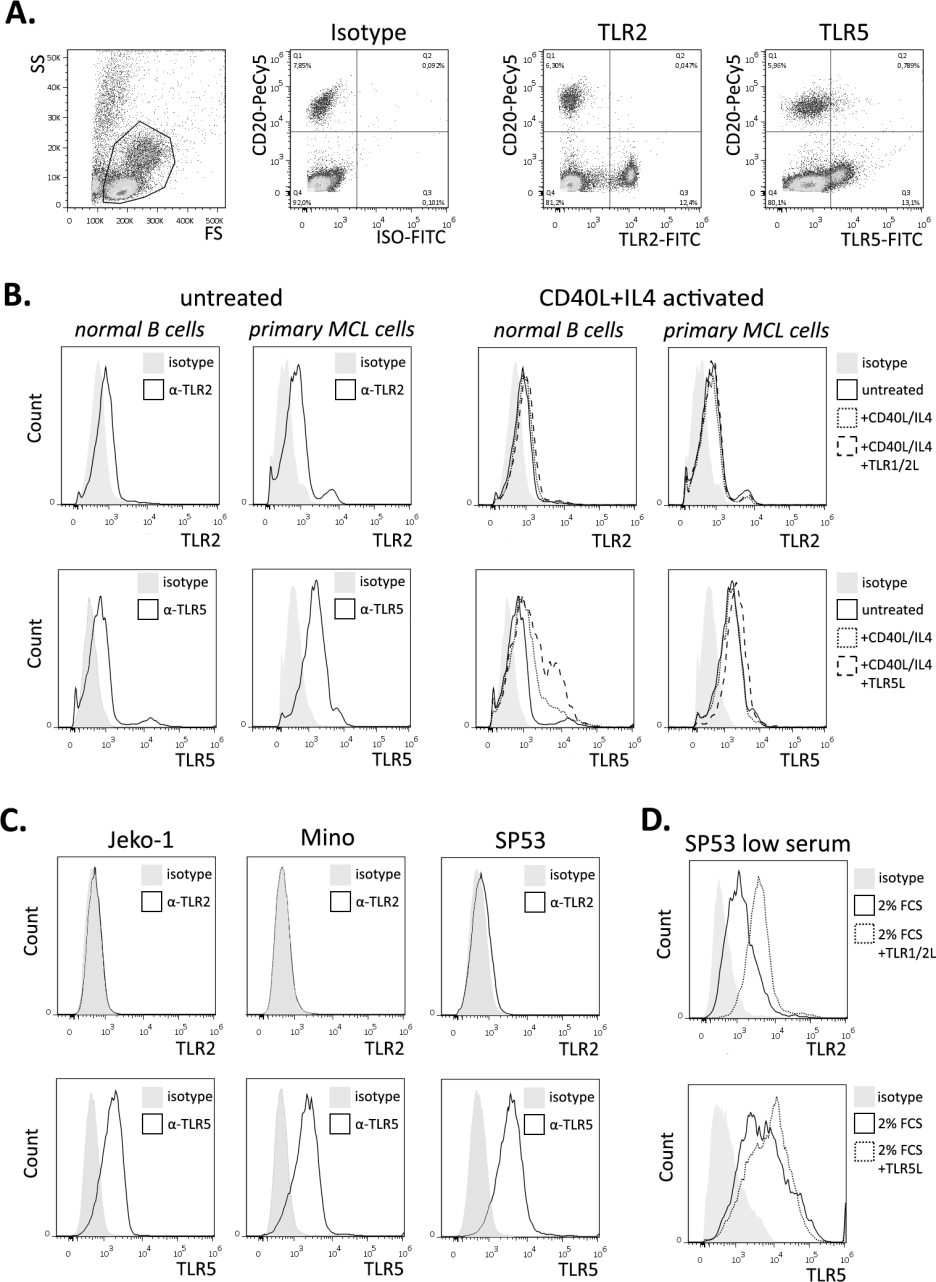
**Analysis of TLR2 and TLR5 expression in normal B lymphocytes and primary MCL cells (A and B), and MCL cell lines (C and D). (A)** Evaluation of TLR2 and TLR5 expression in PBMCs and in CD20+ B cells. (**B)** Monitoring of TLR2 and TLR5 expression in normal B cells and primary MCL cells cultured for 24h in the absence/presence of CD40L, IL-4, and TLR ligands. Filled curves refer to isotypic control, empty curves to the TLR2 or TLR5 antibodies of untreated samples. Dotted histograms represent CD40L+IL4 activated cells, dashed curves indicate CD40L+IL4 activated cells further treated with TLR ligands. (**C)** Evaluation of TLR2 and TLR5 expression in 3 different MCL cell lines. Filled curves refer to isotypic control, empty curves to the TLR2 or TLR5 antibodies. (**D)** Monitoring of TLR2 and TLR5 expression in SP53 cell line cultured under low serum conditions. Filled curves refer to isotypic control, empty curves to the TLR2 or TLR5 expression after 24h at 2% FBS, dotted histograms represent TLR2 or TLR5 expression after 24h in the presence of 2% FBS and TLR1/2 ligand or TLR5 ligand.

Double staining for CD20 and TLR2 or TLR5 carried out in PBMCs from healthy donors showed that circulating B lymphocytes are virtually negative for TLR2 expression, whereas TLR5 is detectable in less than 1% of these cells (Fig 1A). Primary lymphoma cells from a MCL case showed detectable levels of TLR2 and, more markedly, of TLR5 as compared to those of purified normal B lymphocytes (Fig 1B). No evidence of TLR2 up-regulation was observed in normal or MCL cells treated for 24 hours with CD40L and IL-4, whereas this activation enhanced the expression of TLR5 in normal B lymphocytes but did not affect the levels of TLR5 expressed by primary MCL cells (Fig 1B). A further up-regulation of TLR5 was observed in normal B lymphocytes and, to a lesser extent, in MCL cells activated with CD40L and IL-4 and treated with a TLR5 ligand (Fig 1B). No relevant change in TLR2 expression was observed in activated cells after exposure to a TLR2 ligand (Fig 1B). High expression levels of TLR5 were observed in all three MCL cell lines investigated, whereas TLR2 was expressed by SP53 cells and at low-to-undetectable levels in Jeko-1 and Mino cells (Fig 1C). No up-regulation of these receptors was observed after CD40L/IL-4 co-stimulation in MCL cell lines (not shown). A marked up-regulation of TLR2 was observed in SP53 cells cultured for 24 hours under low serum conditions (2% FCS), and a further increase was noticeable when the TLR1/2 ligand was added, whereas the levels of TLR5 increased only marginally (Fig 1D)**.** These data suggest that ligands for TLR2, in particular when forming the heterodimer with TLR1, and for TLR5 may be detected by MCL cells, and potentially have a functional effect on them.

### Triggering of TLR1/2 and TLR5 activates the Akt, MAPK, and NF-κB signaling pathway in MCL cells

**Fig 2:**
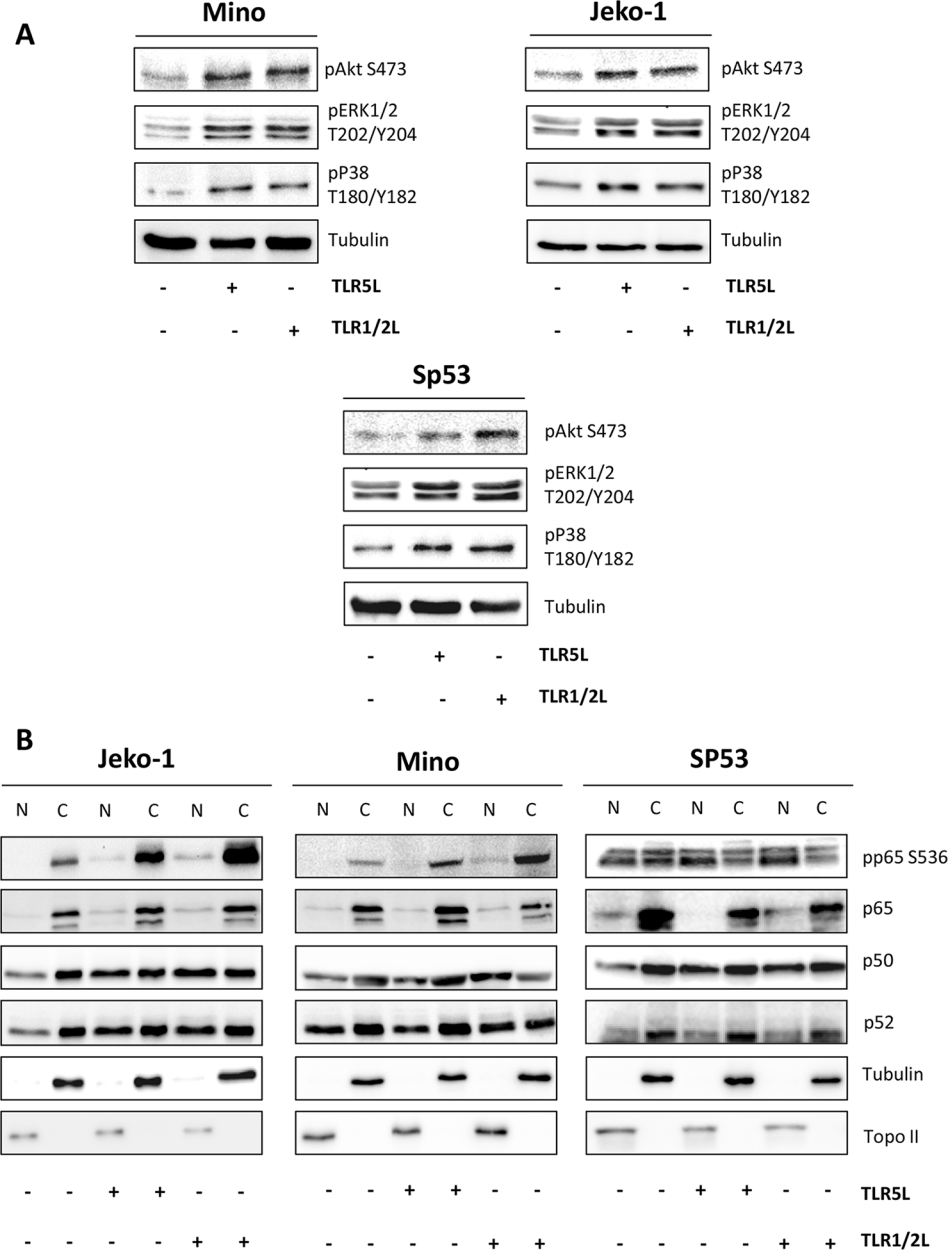
**Triggering of TLR1/2 and TLR5 activates Akt, MAPK (A), and NF-κB pathway (B) in MCL cell lines. (A)** Jeko-1, Mino, and SP53 cell lines were cultured in the absence or presence of TLR ligands (50 ng/ml) for 4 hours. Analysis of Akt, p38, and Erk1/2 phospho-proteins was conducted by immunoblotting with the indicated antibodies. Tubulin shows equal loading of proteins for each lane. **(B)** Nuclear and cytoplasmic fractions for localization of NF-κB complex components were isolated as described in materials and methods. Localization of nucleus-specific protein topoisomerase II (topo II) and cytoplasm-specific protein tubulin was carried out to confirm the purity and equal loading of the nuclear and cytoplasmic fractions.

Given the ability of TLRs to cross-talk with other regulatory pathways [22] we first evaluated the effect of TLRs triggering on the inherent Akt activation and MAPK signaling alteration characterizing this lymphoma.[23,24] As shown in Fig 2A, exposure of MCL cell lines to TLR1/2 or TLR5 ligands enhanced Akt constitutive activation and up-regulated the activating phosphorylation of the MAPK p38 and Erk1/2. Moreover, considering that the NF-κB signaling mediates critical MCL-microenvironment interactions,[25,26] we also investigated the effects exerted by TLR1/2 or TLR5 triggering on this pathway. As shown in Fig 2B, treatment with TLR1/2 and TLR5 ligands enhanced the levels of pp65 (Ser536) in both cytoplasm and nucleus of Jeko-1 and Mino cells. Although SP53 cells showed high basal levels of pp65, particularly in the nucleus, protein phosphorylation was significantly enhanced by TLR1/2 or TLR5 activation also in these cells (Fig 2B). Furthermore, all three cell lines showed increased amount of nuclear p50 after treatment with TLR1/2 or TLR5 ligands (Fig 2B). Analysis of the components of the non-canonical NF-κB pathway revealed increased levels of p52 in the nucleus of Jeko-1 cells after TLR1/2 or TLR5 activation (Fig 2B). No relevant change was observed in the amount and subcellular localization of p52 in Mino and SP53 cells, suggesting a preferential activation of the canonical NF- κB pathway. These findings indicate that TLR1/2 or TLR5 triggering can further de-regulate altered signaling pathways in MCL cells.

### TLR1/2 and TLR5 activation enhance cyclin D1 and D3 expression levels in MCL and primary B cells

**Fig 3:**
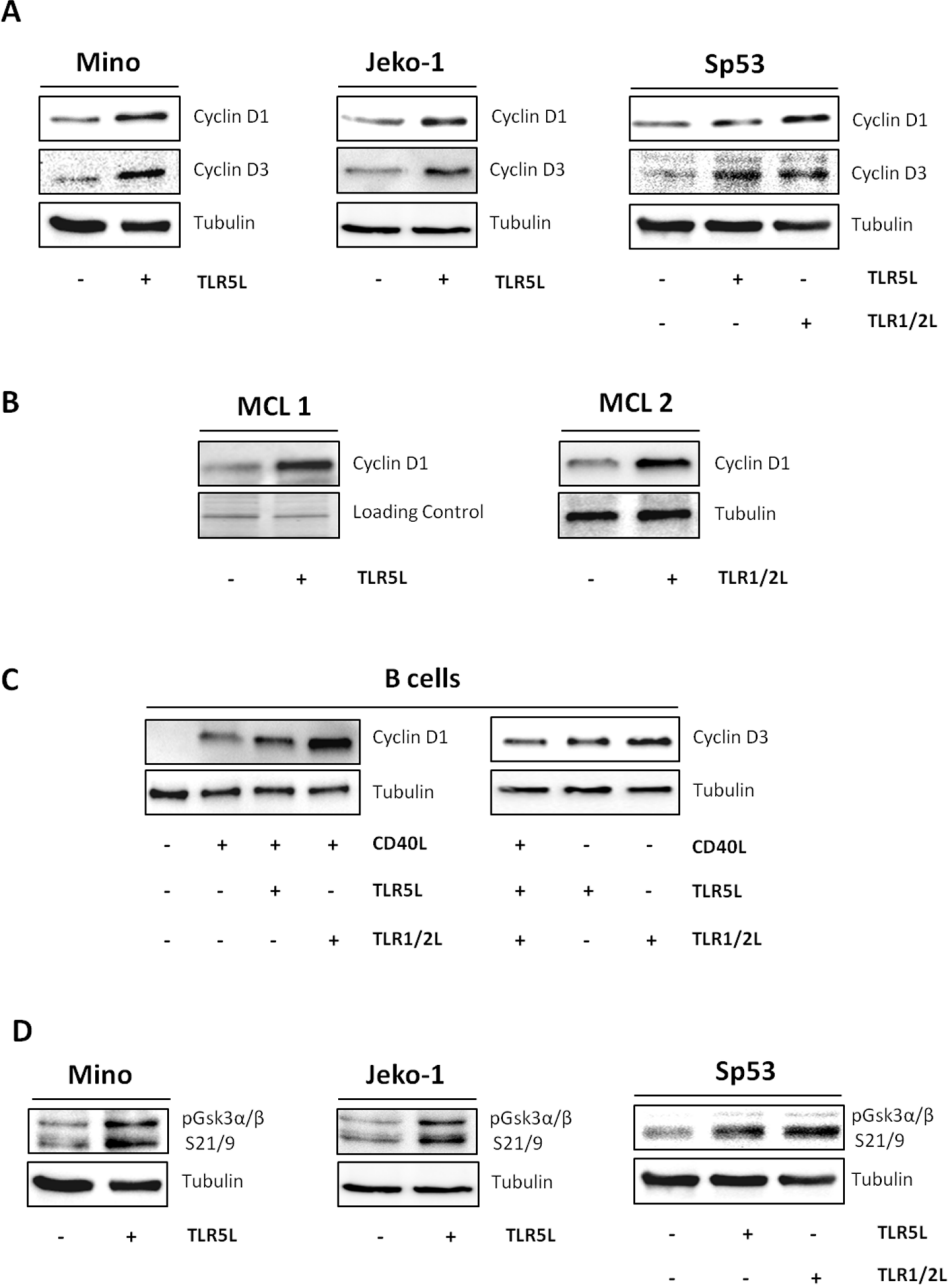
**Exposure to TLR 1/2 and TLR5 ligands enhances cyclin D1 and cyclin D3 expression in MCL cells and activated-B cells from healthy donors (A-C). Cyclins up-regulation is associated with deactivation of GSK-3 α/β kinases (D). (A)** Jeko-1, and Mino cells were exposed to TLR5 ligand (50 ng/ml) for 4 hours, SP53 cells were exposed to TLR5 ligand or TLR1/2 ligand (50 ng/ml) for 4 hours. Protein extracts from untreated and treated cells were used for cyclin D1 and cyclin D3 immunoblotting analysis. Results are representative of three different experiments. **(B and C)** Primary MCL cultures were treated with TLR5 ligand or TLR1/2 ligand (75 ng/ml and 30 ng/ml respectively) for 4 hours (B). B-cells from healthy donors were stimulated with CD40L+IL4+anti-IgM and exposed to TLR5 (75 ng/ml) or TLR1/2 ligand (30 ng/ml) for 4 hours and 72 hours (C). Cyclin D1 and cyclin D3 expression was analyzed by immunoblotting. Tubulin and gel image show equal loading of proteins for each lane. Figures are representative of four different MCL patients and three different B-cells healthy donors. **(D)** Mino and Jeko cells were exposed to TLR5 ligand (50 ng/ml), SP53 cells were exposed to TLR5 ligand or TLR1/2 ligand (50 ng/ml) for 4 hours. Phospho-GSK-3α/β immunoblotting analysis was performed with the indicated antibody.

Akt, MAPK, and NF-κB signaling cascades have a crucial role in regulating cell cycle and proliferation.[27] Based on these evidences, we analyzed the effects of TLR1/2 or TLR5 ligands on the expression of cyclin D1 and cyclin D3 in MCL cell lines and short-term cultures derived from patients with MCL. Immunoblotting analysis disclosed a marked up-regulation of cyclin D1 in all the three TLR5-expressing MCL cell lines investigated (Fig 3A). Cyclin D3 levels were also increased after TLR5 activation in SP53, Mino and Jeko-1 cells (Fig 3A). A similar analysis carried out in the TLR2-expressing SP53 cells showed that also TLR1/2 activation induced cyclin D1 and D3 up-regulation (Fig 3A). The further up-regulation of cyclin D1 expression after TLRs triggering is of particular interest, as the typical cyclin D1 over-expression that characterizes MCL is a necessary but not sufficient condition for lymphomagenesis.[1,2] Noteworthy, the analysis of this protein in four different primary MCL cultures after treatment with TLR1/2 or TLR5 ligands revealed a significant increase of its expression level also in this case (Fig 3B). Interestingly, primary B lymphocytes derived from healthy donors and normally not expressing cyclin D1 showed a marked increase of cyclin D1 expression levels when exposed to CD40L+IL4+anti-IgM, an effect that was even greater when this stimulus was used in combination with TLR1/2 or TLR5 ligands (Fig 3C). Cyclin D3 protein levels were also enhanced by TLR1/2 or TLR5 activation in the same CD40L+IL4+anti-IgM-treated B-cells, after 72 hours of exposure to the ligands (Fig 3C). Conversely, treatment of MCL cells and of stimulated B cells with TLR1/2 or TLR5 ligands did not significantly enhance the mRNA levels of cyclin D1 and D3 assessed by qPCR, ruling out the involvement of transcriptional mechanisms (data not shown).

Along with the enhanced Akt activation, we therefore verified the possible involvement of the Akt downstream kinase GSK-3α/β in the ability of TLRs to modulate the expression of cyclin D1 and cyclin D3. In fact, GSK-3α/β is a critical regulator of cyclin D1 and D3 stability, controlling proteasome-mediated degradation of these proteins at post-transcriptional level.[28–30] As shown in Fig 3D, cyclin D1 and D3 up-regulation induced by TLR1/2 or TLR5 activation was associated to an enhanced phosphorylation and consequent deactivation of GSK-3α/β at Ser21/9, which is known to impinge on cyclins phosphorylation and block their degradation by proteasome.[29,31] These findings demonstrate that the activation of TLR2 and TLR5 in MCL cells has functional effects on key regulators of the cell cycle, such as cyclin D1, most likely involving post-translational mechanisms.

### TLR1/2 or TLR5 activation enhances the proliferation of normal B lymphocytes and MCL cells

**Fig 4:**
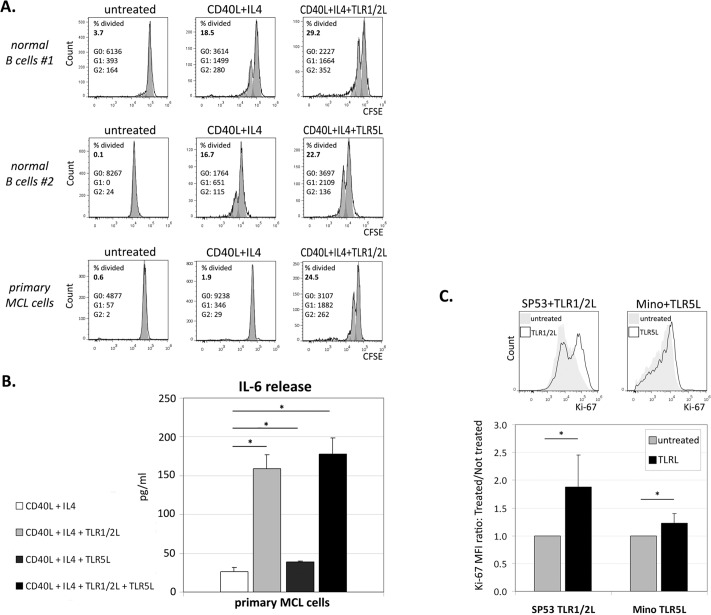
**Effects of TLR ligands on normal B lymphocytes and primary MCL cells proliferation (A), and IL-6 release (B), and evaluation of SP53 and Mino cell lines proliferation in the presence of TLR ligands (C). (A)** Proliferation analyses of CFSE labelled CD40L+IL4 activated B cells (72h) and primary MCL cells (96h), treated with TLR1/2 or TLR5 ligands, obtained from two different healthy donors and one MCL patient. The Percent Divided of each sample, and the number of cells in each generation (G; G0 = not proliferating cells) are indicated. **(B)** IL-6 release in primary MCL cells after activation with CD40L and IL4, and further stimulation with TLR ligands. Histograms represent means and standard deviation of triplicates. IL-6 release in the untreated sample was not detectable. **(C)** Exemplary histogram plots of Ki67 labelling in SP53 and Mino cell lines treated with TLR ligands for 48h. Full curves represent the untreated sample, empty peaks the Ki-67 expression in treated cells. Histogram data are indicated as MFI ratio between treated and untreated samples. Values are calculated as means and standard deviations of 3 independent experiments. * p ≤ 0.05.

Results obtained from cyclin D1 and cyclin D3 analysis prompted us to investigate the effect of TLRs activation on proliferation of normal B cells and MCL cells. Although TLR1/2 or TLR5 triggering with recombinant ligands alone did not affect the proliferation of normal B lymphocytes or primary MCL cells (data not shown), CFSE dilution experiments showed that TLR1/2 or TLR5 ligands were able to increase the number of cell division in normal B lymphocytes activated for 72 hours with CD40L, IL-4 and anti-IgM (Fig 4A). As compared to normal B lymphocytes, primary MCL cells were less responsive to CD40L+IL-4+anti-IgM activation, but they showed a strong increase in the percentage of divided cells when cultured in the presence of TLR1/2 ligand (Fig 4A). Proliferation of primary MCL cells treated with CD40L+IL-4+anti-IgM was not affected by further addition of TLR5 co-stimulation (data not shown). Notably, TLR1/2 triggering significantly enhanced the amount of IL-6 released in the culture supernatant of activated-primary MCL cells (Fig 4B). Treatment of these cells with a TLR5 ligand also increased IL-6 production, albeit to a lower extent. In fact, after the activation with CD40L+IL-4, the TLR5 and TLR1/2 co-stimulation induced an early (after 2 hours) 10-fold increase of IL-6 mRNA (data not shown), but did not significantly enhance the protein levels of IL-6 induced by TLR1/2 triggering (after 96 hours; Fig 4B). CD40L+IL-4 activation of normal B lymphocytes enhanced IL-6 production at such high levels that no further increase induced by TLR1/2 or TLR5 triggering could be documented (data not shown). MCL cell lines showed enhanced proliferation when cultured in the presence of TLR5 or TLR1/2 ligands, as indicated by Ki-67 staining. In particular, SP53 cells were more responsive to TLR1/2 triggering whereas Mino cells showed a more pronounced increase in proliferation when cultured with TLR5 ligand (Fig 4C). These results show that TLR2 and TLR5 ligands could directly contribute to the cell growth of MCL cells and boost the proliferative effects induced by other microenvironmental stimuli, such as CD40L, in B cells, further promoted by the increased production of IL-6.

## Discussion

As for other tumors, a dynamic interaction with the surrounding microenvironment is critical for the growth, survival and immune evasion also of MCL. Indeed, recent evidence suggests a possible concomitant collaboration between TLR and BCR signaling molecules in MCL [32] and indicates that local stimuli able to activate in particular TLR4 or TLR9 may promote MCL cell proliferation and up-regulate the production of immunosuppressive cytokines.[15,16] Despite these findings, the effects mediated by triggering of other TLRs in MCL cells have been poorly investigated.[10] In the present study, we provide evidence that MCL cells may express other functional TLRs of potential pathogenic relevance, particularly TLR2 and TLR5, with a certain degree of heterogeneity. In fact, we noticed detectable levels of TLR2 protein in SP53 MCL cell line as well as in primary MCL cells, whereas TLR5 was expressed by all the three MCL cell lines and primary MCL cell cultures investigated. Moreover, stimulation with CD40L and IL-4 was shown to enhance the levels of TLR5, but not those of TLR2, in normal B lymphocytes, thus suggesting that signals from the germinal center, such as CD40L and IL-4, [33] may render normal B lymphocytes susceptible to the effects mediated by TLR5 activation. As far as MCL cells are concerned, however, it remains to be elucidated whether the aberrant expression of TLR2 and TLR5 is a consequence of malignant transformation or just a reflection of the phenotype of the putative MCL cell precursor, as already described for other types of B-cell malignancies.[34] Furthermore, shortage of growth factors within tumor microenvironment may lead to up-regulation of TLR2 and, to a lesser extent, of TLR5, as shown by culture of MCL cells under low serum conditions. This may probably reflect what may happen *in vivo* as a response to a cellular stress, which may induce the release of endogenous TLR ligands and favor the expansion of TLR-expressing malignant B-cell clones.[10]

The heterogeneous expression of TLRs is also consistent with the highly heterogeneous level of responses to TLR activation observed among different lymphomas and even on a patient-to-patient basis.[10] Consistently, MCL cell lines cultured in the presence of TLR1/2 or TLR5 ligands exhibited enhanced proliferation with a variable extent of response to each stimulus among the cell lines investigated. In particular, the TLR2-expressing SP53 cell line showed increased Ki-67 expression after TLR1/2 triggering, while Mino cell line displayed an increased rate of proliferation after TLR5 ligand treatment. Normal B lymphocytes from different donors also showed a variable response to TLR ligands, which were able to further boost the proliferation induced by a CD40L+IL-4 co-stimulus. As compared with normal B lymphocytes, primary MCL cells were less responsive to the growth-promoting effect of CD40L+IL-4 co-stimulation, but displayed a marked increase in proliferation when cultured in the presence of TLR1/2 ligand. Consistently, CD40L+IL-4 stimulation of primary MCL cells only slightly enhanced IL-6 production, whereas the levels of this cytokine were noticeably increased by TLR1/2 triggering. These effects may be of pathogenic relevance, since IL-6 may promote survival and proliferation of MCL cells in an autocrine/paracrine fashion, as recently demonstrated.[9] The potential relevance of interactions between MCL and the microenvironment has been further enforced through the demonstration that activating mutations of TLR2 triggered secretion of high levels of IL-6 in a subset of MCL. [21] Our findings are also consistent with the need of an additional stimulus to enhance B-cell growth in response to TLR activation.[10] Besides CD40L and IL-4, activation of B-cell receptor (BCR) signaling by cognate antigen may also cooperate with TLR triggering in promoting the proliferation of B lymphocytes.[10,32] In keeping with these data, B-cells obtained from healthy donors in our study increased their growth after TLR1/2 and TLR5 stimulation following activation with the human anti-IgM antibody plus CD40L and IL-4. It is worth mentioning in this respect that MCL cells have a constitutive activation of the BCR signal transduction proteins Syk and PKCβII [35,36] as well as high expression of the phosphorylated forms of these and other BCR-associated kinases.[37] These findings may at least partly explain why MCL cell lines apparently respond with enhanced proliferation to TLR1/2 or TLR5 triggering without the requirement of additional co-stimuli.

One of the most relevant findings of the present study is that the growth-promoting effect exerted by TLR1/2 or TLR5 triggering in MCL cells is associated with cyclin D1 and D3 up-regulation, an effect occurring at post-translational level and likely involving the Akt/GSK-3 axis. In particular, we demonstrate that TLR1/2 or TLR5 triggering enhances the inherent Akt activation of MCL cells,[23] which results in hyperphosphorylation and deactivation of GSK-3, a kinase that critically regulate cyclin D1 and D3 stability.[28,30] Forced inactivation of GSK-3 prevents the phosphorylation of cyclin D1 and D3, an event that is required for the nuclear export and proteasomal degradation of these proteins.[28,30] Notably, nuclear cyclin D1 retention during the S phase was shown to promote genomic instability, further contributing to the malignant evolution of MCL.[38] Our finding that TLR1/2 or TLR5 activation impacts on both cyclin D1 and D3 is of pathogenic relevance in the light of data suggesting that MCL is not exclusively addicted to cyclin D1, which is deregulated by chromosomal translocations, but rather to both cyclin D1 and D3.[38]

Another interesting issue in this context is the finding that proliferative stimuli, including CD40L, IL-4 and anti-IgM, are able to induce cyclin D1 expression in B cells from healthy donors. Even more notable is the fact that the exposure of activated B-cells to TLR1/2 or TLR5 ligands strongly enhances this cyclin D1 induction and increases cyclin D3 protein level. These results are of pathogenic relevance considering that although expressed at mRNA level at extremely low extent,[39] cyclin D1 protein is not regularly expressed in normal B cells, as cell cycle is mostly regulated by cyclin D2 and cyclin D3 in these cells.[40] Moreover, these data suggest that cooperation between a CD40L-rich microenvironment, that characterizes MCL,[41] and a chronic TLR stimulation may have an important role in promoting and/or supporting lymphomagenesis of MCL, where cyclin D1 is constitutively expressed. Strong experimental support to this hypothesis is provided by the demonstration that triggering of TLR1/2 and TLR5 in four different primary MCL cultures significantly up-regulates the expression of the cyclin D1 onco-protein. Interestingly, Gladkikh et al. [39] showed that cyclin D1 mRNA expression level in reactive lymphoid tissue, although lower than in lymphoma samples, is higher than that of normal B lymphocytes, suggesting a step-by-step induction of cyclin D1, culminating with its functional protein expression in tumors, and likely regulated also by microenvironmental factors.

We also provide evidence indicating that TLR1/2 or TLR5 triggering enhances p38 and Erk1/2 activation in MCL cells. Interestingly, constitutive expression and relative involvement of p38 in malignant transformation have been demonstrated in a large number of B-lymphoma-derived cell lines and primary lymphoma tissues.[42] Besides, inhibition of Erk1/2 phosphorylation has been shown to have anti-proliferative and pro-apoptotic activity in different lymphoma cell lines over normal lymphocytes, supporting the role of these MAPKs in lymphomagenesis.[27]

In addition, TLR1/2 or TLR5 engagement promotes functional activity of NF-κB in MCL cells, mainly affecting the canonical pathway. These results suggest that local stimulation of TLR1/2 or TLR5 may contribute to the constitutive activation of NF-κB observed in MCL,[43] behaving thus similarly to the B-cell activation factor BAFF released by stromal cells, which was shown to enhance survival, migration and drug resistance of MCL cells via NF-κB activation.[26]

Altogether, our findings may reflect a local microenvironment characterized by proliferative and pro-inflammatory stimuli, probably deriving from local infection or cellular stress conditions. So far, many lymphoma subtypes have been linked to bacteria and viruses infections. For example, marginal zone lymphoma has been frequently correlated to chronic infections: *Helicobacter pylori* has been involved in gastric MALT-lymphoma, *Chlamydophila psittaci* has been identified in ocular adnexal MALT-lymphoma, whereas *Borrelia burgdorferi* has been detected in cutaneous MALT-lymphoma.[44–46] Intriguingly, a recent large case-control study investigating the association between *Borrelia* infection and risk of non-Hodgkin lymphoma, showed that a previous history of *Borrelia* infection was significantly associated with a nearly 3-fold increased risk of MCL, whether the exposure was self-reported or based on serologic evidence, even in patients with no recollection of *Borrelia* disease.[47] While the implication of *Borrelia* in the development of cutaneous B-cell lymphomas may be explained because of the skin manifestations of borreliosis, the association with noncutaneous lymphomas remains unclear. However, infection with *Borrelia burgdorferi* is not limited to the skin, but also disseminates to other regions including, presumably, the lymphoid tissues.[48] Notably, *Borrelia* DNA was found within the malignant lesions of two patients with nodal B-cell lymphoma, one of which was an MCL.[49] Furthermore, the exposure to European strains of *Borellia burgdoferi* has been associated with increased risk of MCL.[47] Though more in depth mechanistic studies are required, these data provide initial support to the hypothesis that local *Borrelia* infection could be the source of TLR1/2 and TLR5 ligands. Indeed, the Lyme disease-causing bacterium *Borrelia burgdorferi* is endowed with potent ligands for these TLRs, including the outer surface protein A (OspA), a triacylated lipoprotein with strong TLR1/2 stimulatory activity [50] and flagellin, the natural ligand for TLR5.[51–53] The effects of *Borrelia*-derived TLR ligands on TLR2 and TLR5 triggering are still unclear and may depend on cell type. In fact, while in monocytes the activation of TLR2 seems to play the major role in mediating response to *Borrelia*, [50] causing a decrease in TLR5 expression, [54] microglia primary cells show an upregulation in the expression of both TLR2 and TLR5 after *Borrelia* infection. [51,55] Moreover, proteins derived from *Borrelia* flagella are able to both entail an upregulation of TLR5 expression in monocytes, [54] and reduce the expression of the same TLR in mucosal dendritic cells.[56] We need thus to further investigate the influence of the whole bacterium, and not only the derived TLR ligands, on MCL cells, to better understand whether *Borrelia* infection might constitute a microenvironmental factor potentially able to promote MCL cell growth through the potent stimulation exerted by activation of these TLRs, at least in a subset of MCL cases. In perspective, further studies will allow a more thorough understanding on the possible pathogenic association between *Borrelia* infection and MCL.

In conclusion, our results demonstrate that ligands for TLR1/2 or TLR5 may provide critical stimuli able to promote and/or sustain the growth and the malignant phenotype of MCL cells, in particular increasing the expression of cyclin D1 and D3. The identification of microbial and/or endogenous ligands for TLR1/2 or TLR5 may provide new therapeutic targets to be exploited to counteract the tumor promoting effects of lymphoma microenvironment.

## Supporting Information

S1 Tables Raw data for Mean Fluorescence Intensity (MFI) relative to Ki-67 expression in untreated and TLR ligands-treated cell lines. (PDF)
