# Supplementary material for: Toll-Like Receptor 1/2 and 5 Ligands Enhance the Expression of Cyclin D1 and D3 and Induce Proliferation in Mantle Cell Lymphoma
Source: PLoS One. 2016 Apr 28;11(4):e0153823. doi: 10.1371/journal.pone.0153823 (PMC4849792; doi:10.1371/journal.pone.0153823)
Supplement: S1 Tables — (PDF) [file pone.0153823.s001.pdf]

S1.
Supporting information file 1.

Raw data relative to figure 4C

**SP53 with TLR1/2 ligand**	*untreated*		*treated*	
	MFI	*MFI ratio*	*MFI*	*MFI ratio treated/untreated*
Exp1	33.8	1	53.1	1.57
Exp2	18.6	1	41.8	2.25
Exp3	15.3	1	18.9	1.24
Exp4	22.1	1	54.6	2.47
Mean		1		1.88
SD		0		0.57
p-value				***0.02***

**Mino with TLR5 ligand**	*untreated*		*treated*	
	MFI	*MFI ratio*	*MFI*	*MFI ratio treated/untreated*
Exp1	23	1	23.7	1.03
Exp2	5.7	1	7.89	1.39
Exp3	20.8	1	28	1.35
Exp4	17.8	1	20	1.12
Mean		1		1.22
SD		0		0.17
p-value				***0.04***

Mean Fluorescence Intensity (MFI) data relative to Ki-67 expression in untreated and TLR ligands-treated cell lines. Raw data of three independent experiments were reported. Statistical analysis was performed on the MFI ratios obtained from each experiment. The Student’s t test for two-tailed distributions was used for the analysis, and data were considered statistically significant when $p \leq 0.05$ (two-sided). Exp, Experiment; MFI, Mean Fluorescence Intensity; SD, Standard Deviation.
